# Recurrent Superenhancer of the Oncogene *POU5F1B* in Colorectal Cancers

**DOI:** 10.1155/2021/5405060

**Published:** 2021-12-11

**Authors:** Han-chuan Tao, Cheng Wang, Ning Ma, Xun Zhu, Xiao-jun Zhou

**Affiliations:** ^1^Department of General Surgery, Dongtai Municipal People's Hospital of Nantong University, Yancheng, 224200 Jiangsu, China; ^2^Department of General Surgery, First Affiliated Hospital of Soochow University, Soochow, Jiangsu, 215006 Jiangsu, China; ^3^Department of Neurology, Dongtai Municipal People's Hospital of Nantong University, Yancheng, 224200 Jiangsu, China; ^4^Department of General Surgery, Daqing Oilfield General Hospital, Daqing, 163001 Heilongjiang, China

## Abstract

Superenhancer usages in single cancer form such as colorectal cancer (CRC) may provide novel efficient targeting candidates. It is unclear whether CRC contains recurrent superenhancers that confer a predisposition to malignancy. We investigated the superenhancer profile of CRC cell line HCT116 and compared it to that of a healthy sigmoid colon. We found that HCT116 had lost most of the normal colon superenhancer activities but gained a new set of tumor-favoring superenhancers that facilitate tumor proliferation, growth signalling, and hypoxia resistance. Inhibiting the superenhancers by JQ-1 treatment had significantly decreased the colony formation capability of HCT116. Then, by comparing the superenhancer genes and robust CRC upregulated genes, we identified a superenhancer associated with a common CRC upregulated oncogene, *POU5f1B*. *POU5f1B* overexpression is related to the worse outcome in CRCs. Via performing ChIP-PCR in 35 clinical samples and investigating CRC anti-H3K27ac ChiP-seq public dataset consisting of 36 samples, we further identified that the superenhancer of oncogene *POU5F1B* is recurrently activated in CRCs, taking 62 and 72 per cent, respectively. Moreover, JQ-1 treatment successfully inhibited the POU5F1B expression in 5 out of 6 *POU5F1B* superenhancer-positive samples. Therefore, we concluded that the superenhancer activation of *POU5F1B* contributes partially to its high expression in CRCs, in addition to the well-known gene amplification aetiology.

## 1. Introduction

Colorectal cancer (CRC), or colorectal carcinoma, is the third most common neoplasm and the second leading cause of cancer death worldwide, accounting for 1.8 million cases and 862,000 deaths in 2018 [1]. CRC often arises from precancerous states such as polyp and accumulates several aberrant oncogenic and tumor-suppressive drivers to claim malignancy. The deterioration proceedings may involve genomic mutations and epigenetic alterations, some of which are noncoding regulatory elements like superenhancers [2–4]. Pelish et al. initially brought up the superenhancer concept when his team first noticed the clusters of enhancers spanning several kilobases with proximity to or overlapping the gene bodies of the pluripotent transcription factors *Oct4*, *Nanog*, and *Sox2* in mouse embryonic stem cells (ESC) [2]. Moreover, they observed that the superenhancers usually guard identity gene expressions in various cell types [3, 4]. Previous studies reported that CRC and other cancer forms usually accompany widespread locus-specific enhancer activity dysregulations [5–7]. As cancer and stem cells have many convergent gene expression modules, they resemble adopting superenhancers to drive the potency or oncogenic transcriptional activities [8]. In vitro tumor model assays revealed that leading compounds targeting superenhancer assembly or activation had yielded promising tumor growth reductions [9, 10]. Therefore, investigating superenhancer usages in single cancer forms such as CRC may provide novel targeting candidates.

To date, researchers have carried out epigenetic profiling on substantive cancer forms, including CRC, for cisregulatory element annotations [11–13]. Among them, there are several types, including anti-H3K27ac ChIP-Seq, anti-H3K4me1 ChIP-Seq, and DNase-Seq, applicable for superenhancer profiling analyses. However, few studies have interrogated CRC superenhancer landscapes comparing to normal colons. Consequently, there is limited recognition of tumor drivers composed of superenhancers in CRC. Despite the heterogeneous nature of tumors, it is unclear whether CRC contains recurrent superenhancers that confer a predisposition to malignancy similarly to the genomic significantly mutated hotspots.

We investigated the superenhancer profile of CRC cell line HCT116 and compared it to a healthy sigmoid colon. We found that the HCT116 had lost most of the normal colon superenhancer activities but gained a new set of tumor-favoring superenhancers with the critical transcription factors of *KLF4*, *MAZ*, *MYC*, and *SMAD3*, forming self-rewiring transcriptional regulations that facilitate tumor proliferation, growth signalling, and hypoxia-resistant transcription factors. Moreover, we identified that the superenhancer of oncogene *POU5F1B* is recurrently activated in CRCs. The superenhancer activation of *POU5F1B*, adding the gene body amplification, consists of the significant aetiology for the specific high expression of *POU5F1B* in CRCs.

## 2. Materials and Methods

### 2.1. Public Datasets

We obtained the HCT116 and sigmoid colon anti-H3K27ac ChIP-Seq raw sequencing reads from ENCODE with the accession code of ENCFF225QAB and ENCFF898XDY. Human CRC and cell line COLO205 anti-H3K27ac ChIP-Seq and RNA-Seq processed results were obtained from the GEO dataset with the accession code of GSE77737 [13].

### 2.2. H3K27ac ChIP-Seq Analysis and Superenhancer Identification

ChIP-seq data were aligned to the hg19 genome assembly, using Bowtie 2 v2.0.6 [14], discarding reads with at least one mismatch and reporting the best alignment if multiple alignments were present. PCR duplicates were removed using SAMtools [15, 16]. Peaks were detected with MACS2 [17, 18] with default parameters. Peak lists were filtered to remove all peaks overlapping ENCODE blacklisted regions. Superenhancer was examined by ROSE [10, 18] with default settings.

### 2.3. Gene Ontology (GO) and PPI Analysis

GO and PPI analyses for superenhancer-related gene lists were performed on genes associated with superenhancers using Metascape [19]. FDR cut-off was <0.05.

### 2.4. Motif Enrichment Analysis

We used rgt-hint [20] to perform the motif enrichment analysis. Briefly, we firstly implied the potential TF binding sites via footprinting analysis of the superenhancer regions. Then, we performed motif scanning and statistical enrichment analysis on the footprints.

### 2.5. Cell Culture. Transwell and Colony Formation Assay

Cells were cultured in DMEM supplemented with 10% fetal bovine serum (FBS) (ThermoFisher Scientific) and 100 *μ*g/ml penicillin-streptomycin-glutamine (ThermoFisher Scientific) at 37°C with 5% CO_2_ in a humidified incubator. For colony formation assay, 10^3^ cells were plated onto 35 mm dishes in triplicate and cultured for about one week. Cells were rinsed with cold PBS, fixed with ice-cold methanol, and stained with Coomassie brilliant blue. For transwell assay, Costar transwell cell culture inserts (Corning) were used for cell culture for 24 h, starting with a layer of 5 × 10^4^ cells. Then, we fixed the cells with ice-cold methanol and stained with 0.2% crystal violet. Migration was evaluated by counting the number of cells migrating to the other side. Experiments were performed in triplicate.

### 2.6. RT-qPCR

RT-qPCR for *POU5F1B* was performed. The forward and reverse primers are listed in supplementary table 2. According to the manufacturer's instruction, total RNAs were extracted from cells with TRIzol (ThermoFisher Scientific) and cDNAs were synthesized using reverse transcription kit (Takara). Real-time PCR was performed using SYBR Green Master Mix (Takara) and ABI StepOnePlus system (ThermoFisher Scientific). *GAPDH* mRNA was examined as an internal control.

### 2.7. ChIP-qPCR

Anti-BRD4 and anti-H3K27ac Chip-qPCR of POU5F1B superenhancer was performed, respectively. The forward and reverse primers are listed in supplementary table 2. Chromatin immunoprecipitation (ChIP) assay was performed according to the protocol developed by Upstate Biotechnology. Briefly, chromatin lysates were prepared from hepatocytes after crosslinking with 1% formaldehyde. The samples were precleared with Protein-G agarose beads and immunoprecipitated using anti-H3K27ac antibody (Abcam, #cat: ab4729), anti-BRD4 antibody (Abcam, #cat: ab128874), or control anti-IgG (Abcam, #cat: ab172730) in the presence of BSA. Beads were extensively washed before reverse crosslinking. DNA was purified using a PCR Purification Kit and subsequently analyzed by qPCR.

### 2.8. Western Blotting

Cells were lysed in lysis buffer (1% Nonidet P-40 and 0.1% SDS in 1× PBS solution supplemented with proteinase inhibitors). Then, we extracted protein from cultured cells and then subjected 50 mg per sample to SDS/PAGE and transferred to nitrocellulose membrane. To block nonspecific binding, the membrane is placed in a dilute solution of nonfat dry milk. Then, we incubated anti-POU5F1B (Abcam, #cat: ab230429) antibodies or anti-*β*-actin (inner control) (Abcam, #cat: ab179467) as first antibody and then incubate with secondary antibody (Abcam, #cat: ab179467) to visualize by ProteinSimple system (ProteinSimple) (Abcam, #cat: ab288151).

### 2.9. SiRNA Knockdown

We used RNAi-Ready pSIREN-RetroQZsGreen vector (Clontech) for siRNA targeting POU5F1B. A blank vector was used as control. Two siRNA sequences were used; they were 5′-CATACGGTCACAGAGCTTTTT-3′ and 5′-AACTAGTATAGATCGATTCTT-3′, respectively.

## 3. Results

### 3.1. HCT116 Adopts Addiction on Tumor-Favoring Superenhancers

**Figure 1:**
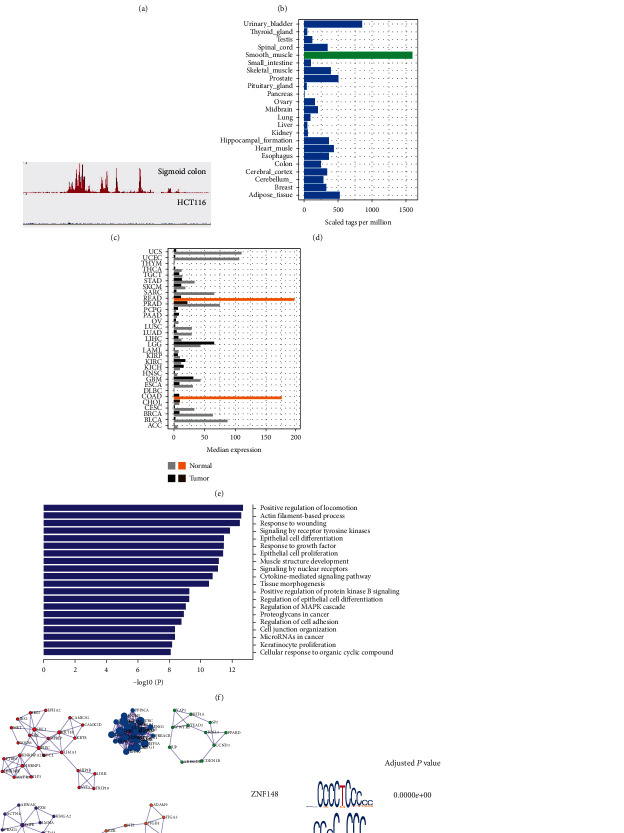
Comparison of the superenhancer profiles of HCT116 and sigmoid colon tissue: (a) heat map of the superenhancers; (b) Venn diagram of the superenhancers of HCT116 and sigmoid colon tissue; (c) the H3K27ac peak enrichment at the superenhancer of *SORBS1* in the sigmoid colon tissue; (d) tissue distribution of the expression of *SORBS1*; (e) *SORBS1* expression in the tumor/tissue of different cancers; (f) GO enrichment of the HCT116 superenhancer genes; (g) gene association module of the GO enrichments in (f); (h) the motifs of overrepresented TFs in the HCT116 H3K27ac superenhancer peaks; (i) JQ-1 inhibits HCT116 colony formation.

To interrogate CRC superenhancer landscape compared to the healthy colon, we first performed ROSE (rank ordering superenhancers) analysis to identify superenhancers via anti-H3K27ac ChIP-Seq data of HCT116, which is a CRC cell line, and normal sigmoid colon tissue. We obtained 32 and 570 (Supplementary Table 1) exclusive superenhancers in HCT116 and the sigmoid colon, respectively (Figure 1(a)). The average length of the superenhancers was 7.8 kb. As shown in the heat map, the HCT116 exclusive superenhancers had acquired more abundant H3K27ac histone modifications in these regions (Figure 1(b)).

To validate that these superenhancers are cell identity related, we examined the superenhancers with the most intensive H3K27ac activities in the control tissue. The highest-ranking superenhancer matches gene *SORBS1* (Figure 1(c)). It encodes an adaptor protein that regulates cell adhesion, growth factor signalling, and cytoskeletal formation. Further human tissue distribution profiling illustrated that *SORBS1* is abruptly high expressed in the smooth muscle and colon (Figures 1(d) and 1(e)). Subsequent checking of the top-ten superenhancers encompassed similar colon- and smooth muscle-specific genes (Supplementary Table 1). The result suggests that superenhancers' healthy sigmoid colon tissue contributes to its cell identity.

Given that HCT116 had acquired more (574 versus 36) and a different set of superenhancers, we performed pathway enrichment analysis to see if they favored tumor proliferation. It revealed that the HCT116 exclusive superenhancer regulated genes enriched in the receptor tyrosine signalling pathway, cell proliferation, epithelial regulation, and cancer miRNA pathways (Figure 1(f)). Further PPI (protein-protein interaction) enrichment indicated seven overpresented PPI sets (Figure 1(g)), whose functions largely reconcile with the most enriched GO terms. Moreover, the HCT116 superenhancer loci overrepresented TF motifs of ZNF148, KLF5, and MAZ (Figure 1(h)). These Zinc finger TFs mainly function in promoting cell proliferation. MAZ is a hypoxia tolerance induction TF, which would be required substantially in the colon and rectal tumors. Besides, EWSR1-FLI1 and FOSB::JUNB were also enriched (Figure 1(h)).

Next, we treated HCT116 with JQ-1, which blocks superenhancer formation via inhibiting the function of *BRD4*. After 12 hours of the JQ-1 exposure, we examined the top-five superenhancer-regulated genes. They were consistently downregulated two- to fivefold. Besides, the colony formation capabilities of HCT116 decreased by 72 per cent (Figure 1(i)).

Altogether, the data indicated that HCT116 abandoned most of its original cell identity superenhancers as the healthy sigmoid colon. Instead, they acquired a broader set of new superenhancers that upregulate gene expressions that enrich tumor-favoring pathways and substantially contribute to tumor growth.

### 3.2. Superenhancer-Activated *POU5F1B* Facilitates Tumor Progression

**Figure 2:**
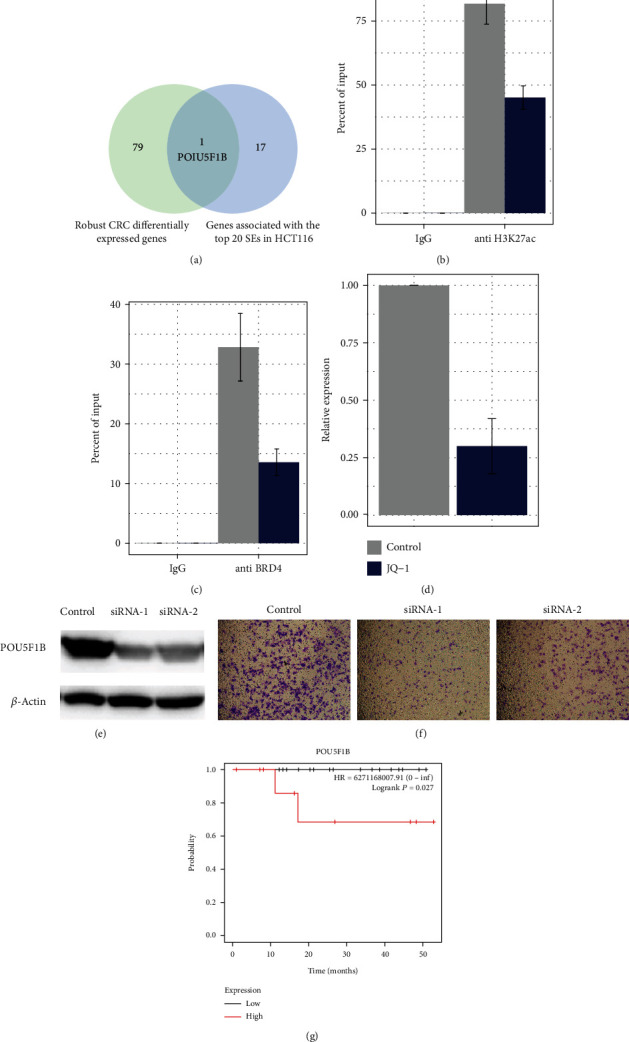
HCT116 superenhancers overlapped with robust CRC genes at POU5F1B. (a) Venn diagram of robust CRC DEGs (differentially expressed genes) and the genes associated with the top 20 SEs (superenhancers) in HCT116; (b, c) COLO205 Chip-qPCR of anti-H3K27ac and BRD4 of *POU5F1B* with or without JQ-1 treatment; (d) COLO205 qPCR of POU5F1B with or without JQ-1 treatment; (e) Western blot siRNA knocking down effect obvious in the protein level; (f) transwell assay showing that migration of the tumor cells was reduced after POU5F1B downregulation; (g) the high *POU5F1B* expression group had worse overall survival.

To obtain the robust and recurrent gained superenhancers in CRC, we intersected the HCT116 superenhancers with another CRC cell line, COLO205, and found their highest twenty common superenhancers. Then, we aligned their corresponding genes to the most robust CRC transcriptionally elevated genes identified through 11 CRC cohorts [21]. Fortunately, the two gene lists have one overlap, *POU5F1B*, a pseudogene of the pluripotency transcription factor *POU5F1*, or *OCT4* (Figure 2(a)).

Next, we investigated if this candidate superenhancer is responsible for *POU5F1B* gene activation in the two cell lines. In the published RNA-Seq expression profiles, *POU5F1B* was highly expressed in COLO205 but barely expressed in HCT116, compared to regular tissue expression. The HCT116 promoter may have been repressed and hitherto maintain the gene in silence despite a superenhancer formation. We then tested the H3K27ac and BRD4 status via ChIP-PCR targeted to this region in the CRC cell line COLO205. The treatment significantly depleted both BRD4 and H3K27ac modifications within the region (Figures 2(b) and 2(c)). Then, we treated COLO205 with JQ1; it significantly inhibited the POU5F1B gene expression by 2.7-folds (*P* value = 5*e*-4; Wilcoxon test) (Figure 2(d)). The results suggested that the superenhancer formation contributed to the high expression of oncogene *POU5F1B* in COLO205 but not in HCT116.

**Figure 3:**
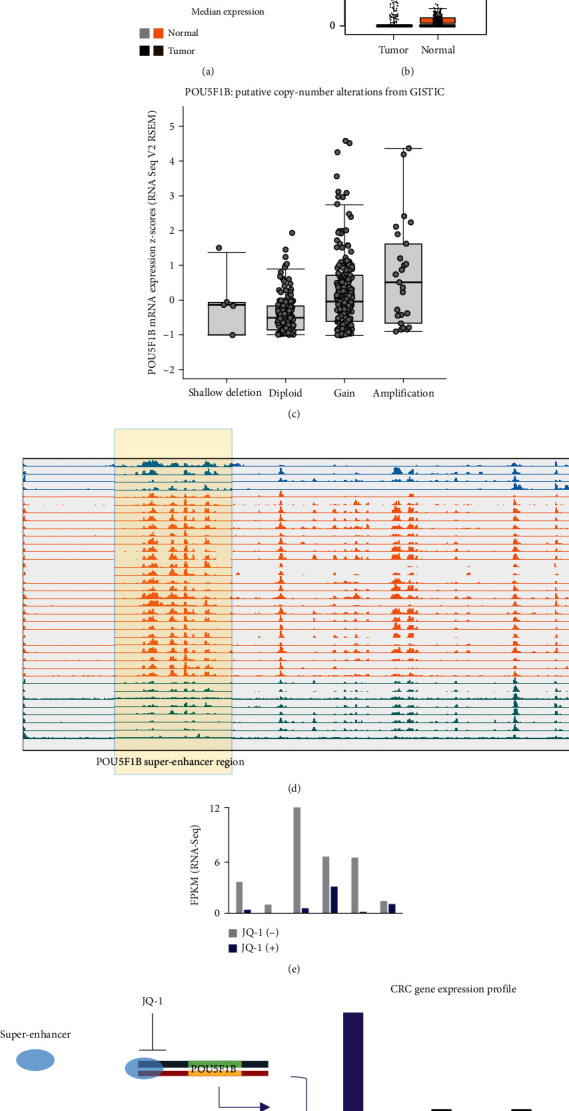
POU5F1B superenhancer is recurrent in CRC. (a) *POU5F1B* expression is abruptly high in the tumors of colorectal cancers (READ and COAD); (b) *POU5F1B* in the tumor and normal tissues in CRC; (c) high expression of *POU5F1B* also occurs in the shallow deletion and diploid and copy number gain groups; (d) *POU5F1B* superenhancer is recurrent in human CRC samples; (e) JQ-1 inhibited *POU5F1B* expressions in human CRC-derived cell culture, which had positive *POU5F1B* superenhancers; (f) diagram showing that superenhancer activation of *POU5F1B* is recurrent in CRCs.

Then, we knocked down the *POU5F1B* expression in COLO205. We achieved an obvious knocking down effect at a protein level (Figure 2(e)). We noticed significant decrease of colonies in three random selected fields (*P* value = 5*e*-7) for the transwell migration assay. Furthermore, we found that the gene expression of *POU5F1B* predicted worse overall survival in early-stage CRCs (Figure 3(c)).

### 3.3. Recurrent *POU5F1B* Superenhancer Activation Contributes to Its High Expression in CRC

Looking at the pancancer expression level of *POU5F1B*, we observed that the gene is explicitly highly expressed in colon CRCs comparing to normal tissues (*P* value < 1.62*E*-12; Wilcoxon test), including COAD and READ (Figures 3(a) and 3(b)). It is well-studied that copy number amplification is a significant factor contributing to the upregulation of POU5F1B in several cancers. In The Cancer Genome Atlas (TCGA) cohort of CRC, we observed 6 per cent of *POU5F1B* copy number amplification in the cancer genomes. Meanwhile, the DNA copy number and mRNA abundance present a positive correlation (Figure 3(c)). However, in TCGA cohort of CRC, 20 per cent of the tumor samples had higher expressed *POU5F1B*, while the amplification recurrence of *POU5F1B* gene bodies in CRC accounts for only 10 per cent. Therefore, some other underlying mechanisms might exist for its upregulation as *POU5F1B* copy number amplification does not cover all the highly expressed cases.

Therefore, novel *POU5F1B* superenhancer formation, as observed in both the HCT116 and COLO205, could be another underlying event contributing to its specific high expression. To test if the gained superenhancer of *POU5F1B* is recurrent in CRC, we collected 36 clinical samples of CRC and assigned them into *POU5F1B* highly expressed group (*n* = 15) and the baseline group (*n* = 21). Then, we used anti-H3K27ac ChIP-qPCR to test the corresponding HCT116 superenhancer interval (Supplementary Table 1). We found that 62 per cent of the highly expressed group had more than 5-fold signal enrichment than in the baseline group. Moreover, we analyzed another anti-H3K27ac ChIP-Seq dataset which includes 36 CRC cell lines and clinical samples. The result revealed a surprising consistency of *POU5F1B* superenhancer in 72 per cent of the samples (Figure 3(d)). Besides, we obtained RNA-Seq data or 6 POU5F1B superenhancer positive CRC samples, with data both in the conditions of pre- and post-JQ-1 treatment. It revealed that the JQ-1 treatment had inhibited more than 90 per cent of the *POU5F1B* expression in 4 of the samples (Figure 3(e)).

The data collectively indicated that *POU5F1B* superenhancer adoption is a recurrent event in CRC that contributes to the oncogenic high expression.

## 4. Discussion

This study found that the healthy sigmoid colon has cell identity gene activating superenhancers, whereas the HCT116 as a CRC cell line has lost most of it and forms an addiction to a new repertoire of tumor-favoring superenhancers. The superenhancers contribute to the high expression of tumor-associated genes like *Myc* and *SMAD3*. Moreover, it is interesting to mention that the TF motifs enriched in the superenhancer regions overlapped with the superenhancer genes, with *MYC* and *KLF5* becoming self-wiring in transcriptional regulations. Therefore, the cell might have formed a positive closed loop to sustain cell growth and proliferation signalling. Additionally, the TFs and genes inferred with substantial cross talks described in previous studies [22–24]. Therefore, via the superenhancers on these TFs, the HCT116 continuously exerts the gene expressions that deposit the cells for proliferation and tolerating extreme hypoxia stress. These superenhancers, especially those that activate master TFs, could undoubtedly provide more evidence supporting the well-studied notion that tumor cells may become addicted to a new set of superenhancers that benefit the tumor cells.

Despite the master TFs, we noticed a prevalent CRC upregulated oncogene, POU5F1B. Researchers initially identified POU5F1B as a pseudogene with no particular function. However, further studies confirmed that it has a particular activity similar to the pluripotency marker *POU5F1B* and may play a role in carcinogenesis and eye development. Previous studies proved that *POU5F1B* promotes tumor growth and aggressiveness in several cancers, including gastric cancer and CRC [25–27]. The well-studied mechanism for its high-incidence upregulation in these cancers is gene amplification. However, the gene amplification percentage is only about half the *POU5F1B* upregulated CRC cases which indicates other mechanisms. Our study's superenhancer contribution findings have explicitly supplemented the understanding of how *POU5F1B* is prevalently activated in CRCs (Figure 3(f)).

In conclusion, through CRC superenhancer investigations, we identified that the superenhancer activation of oncogene *POU5F1B* is prevalent in CRC, contributing to the oncogenic gene upregulated expressions.

## Data Availability

The data used to support the findings of this study are included within the supplementary table.

## Supplementary Materials

Supplementary 1 Supplementary Table 1: superenhancer gene list of HCT116 and normal sigmoid colon.

Supplementary 2 Supplementary Table 2: PCR primers.
