# Supplementary material for: Recurrent Superenhancer of the Oncogene POU5F1B in Colorectal Cancers
Source: Biomed Res Int. 2021 Dec 11;2021:5405060. doi: 10.1155/2021/5405060 (PMC8684575; doi:10.1155/2021/5405060)
Supplement: Supplementary 1 — Supplementary Table 1: superenhancer gene list of HCT116 and normal sigmoid colon. [file 5405060.f1.pdf]

| Supplementrary Table 1. Super-enhancers of HCT116 and sigmoid colon tissue | | | | |
|---|---|---|---|---|
| CHROM | START | STOP | OVERLAP_GENES | sample |
| chr4 | 30715635 | 30985709 | PCDH7 | HCT116 |
| chr12 | 76012567 | 76308058 | | HCT116 |
| chr8 | 94806886 | 94974157 | PDP1,TMEM67 | HCT116 |
| chr1 | 8062403 | 8276419 | ERRFI1 | HCT116 |
| chr17 | 57819217 | 57948130 | TUBD1,MIR21,TMEM49 | HCT116 |
| chr8 | 128213237 | 128320517 | | HCT116 |
| chr18 | 3583398 | 3671591 | FLJ35776,DLGAP1 | HCT116 |
| chrX | 45556718 | 45712940 | MIR221,MIR222 | HCT116 |
| chr9 | 21504387 | 21620428 | MIR31,LOC554202 | HCT116 |
| chr8 | 126648569 | 126715409 | | HCT116 |
| chr7 | 130577088 | 130613286 | LOC646329 | HCT116 |
| chr2 | 226962857 | 227081710 | | HCT116 |
| chr10 | 95066150 | 95240291 | MYOF | HCT116 |
| chr4 | 169396470 | 169587808 | PALLD,DDX60L | HCT116 |
| chr2 | 227474323 | 227667763 | IRS1 | HCT116 |
| chr12 | 66211168 | 66360711 | HMGA2,RPSAP52 | HCT116 |
| chr4 | 75533644 | 75609159 | | HCT116 |
| chr1 | 66720417 | 66842146 | PDE4B | HCT116 |
| chr12 | 65815524 | 65960302 | MSRB3 | HCT116 |
| chr3 | 98230313 | 98310800 | CPOX,CLDND1,GPR15 | HCT116 |
| chr1 | 94626600 | 94801626 | ARHGAP29 | HCT116 |
| chr10 | 114273809 | 114301008 | VTI1A | HCT116 |
| chr12 | 89311073 | 89367919 | | HCT116 |
| chr8 | 126120895 | 126252220 | NSMCE2 | HCT116 |
| chr6 | 151325928 | 151463163 | MTHFD1L | HCT116 |
| chr15 | 67313619 | 67489329 | SMAD3 | HCT116 |
| chr20 | 52719281 | 52796961 | CYP24A1 | HCT116 |
| chr4 | 11586609 | 11655306 | | HCT116 |
| chr8 | 144978519 | 145051891 | PLEC,PARP10,MIR661 | HCT116 |
| chr12 | 65994430 | 66093741 | | HCT116 |
| chr11 | 118758450 | 118819179 | CXCR5,BCL9L | HCT116 |
| chr9 | 21666769 | 21713247 | | HCT116 |
| chr10 | 97067908 | 97329002 | SORBS1 | sigmoid col |
| chr8 | 30239370 | 30463132 | GTF2E2,RBPMS | sigmoid col |
| chr2 | 218593831 | 218869769 | TNS1,DIRC3 | sigmoid col |
| chr10 | 80819113 | 81097206 | ZMIZ1,LOC283050 | sigmoid col |
| chr7 | 134361432 | 134652706 | BPGM,CALD1 | sigmoid col |
| chr2 | 240119391 | 240322816 | HDAC4,MIR4269 | sigmoid col |
| chr14 | 69307184 | 69453786 | ACTN1 | sigmoid col |
| chr2 | 238026837 | 238331022 | COL6A3 | sigmoid col |
| chr22 | 36673350 | 36786955 | MYH9 | sigmoid col |
| chr1 | 39541741 | 39902274 | KIAA0754,MACF1 | sigmoid col |
| chr12 | 124836892 | 125054594 | NCOR2 | sigmoid col |
| chr17 | 75345538 | 75491773 | SEPT9,MIR4316 | sigmoid col |
| chr2 | 206544923 | 206676527 | NRP2 | sigmoid col |
| chr4 | 119808810 | 119968619 | SYNPO2 | sigmoid col |
| chr9 | 124022694 | 124138630 | STOM,GSN | sigmoid col |
| chr16 | 15790669 | 15974706 | NDE1,MYH11,C16orf63 | sigmoid col |
| chr19 | 3332873 | 3470677 | NFIC | sigmoid col |

| | | | | |
|---|---|---|---|---|
| chr8 | 38584294 | 38824118 | TACC1,PLEKHA2 | sigmoid col |
| chr11 | 12381536 | 12579807 | PARVA | sigmoid col |
| chr12 | 11800965 | 12039289 | ETV6 | sigmoid col |
| chr14 | 68541323 | 68777621 | RAD51L1 | sigmoid col |
| chr1 | 10688467 | 10857232 | CASZ1,PEX14 | sigmoid col |
| chr21 | 47370259 | 47563978 | FTCD,COL6A1,COL6A2 | sigmoid col |
| chr2 | 66651643 | 66814445 | MEIS1 | sigmoid col |
| chr2 | 220267587 | 220352786 | DES,SPEG | sigmoid col |
| chr1 | 226814470 | 226929055 | ITPKB | sigmoid col |
| chr3 | 66391598 | 66558774 | LRIG1,SLC25A26 | sigmoid col |
| chr19 | 13106553 | 13216371 | TRMT1,NFIX,LYL1 | sigmoid col |
| chr17 | 46616216 | 46679458 | HOXB4,HOXB6,LOC404266,H( | sigmoid col |
| chr4 | 7744682 | 7981377 | ABLIM2,AFAP1,AFAP1-AS1 | sigmoid col |
| chr1 | 22179166 | 22288972 | HSPG2 | sigmoid col |
| chr10 | 88420951 | 88609766 | LDB3,OPN4,BMPR1A | sigmoid col |
| chr1 | 19667678 | 19861084 | CAPZB | sigmoid col |
| chr17 | 735217 | 884454 | NXN | sigmoid col |
| chr9 | 94527617 | 94717610 | ROR2 | sigmoid col |
| chr11 | 19617115 | 19869182 | NAV2,LOC100126784 | sigmoid col |
| chr3 | 57984099 | 58163937 | FLNB | sigmoid col |
| chr16 | 16039874 | 16254980 | ABCC1,ABCC6 | sigmoid col |
| chr3 | 123324340 | 123443391 | MYLK | sigmoid col |
| chr9 | 117777933 | 117931898 | TNC,DEC1 | sigmoid col |
| chr3 | 73520110 | 73683862 | PDZRN3 | sigmoid col |
| chr3 | 71005546 | 71194823 | FOXP1 | sigmoid col |
| chr17 | 55962597 | 56034365 | CUEDC1 | sigmoid col |
| chr1 | 203592431 | 203696209 | ATP2B4 | sigmoid col |
| chr14 | 74177567 | 74265026 | C14orf43,PNMA1 | sigmoid col |
| chr1 | 27800771 | 27931312 | WASF2,AHDC1 | sigmoid col |
| chr14 | 92977376 | 93161366 | RIN3 | sigmoid col |
| chr5 | 58364186 | 58625634 | PDE4D | sigmoid col |
| chr20 | 42726202 | 42841796 | JPH2,C20orf111 | sigmoid col |
| chr10 | 71151703 | 71268339 | HK1,TSPAN15,TACR2 | sigmoid col |
| chr1 | 8401601 | 8538907 | SLC45A1,RERE | sigmoid col |
| chr16 | 70672684 | 70794789 | LOC100130894,VAC14,MTSS1 | sigmoid col |
| chr10 | 81134663 | 81214794 | ZCCHC24 | sigmoid col |
| chr18 | 42258308 | 42440606 | SETBP1 | sigmoid col |
| chr5 | 156874035 | 157004958 | NIPAL4,ADAM19 | sigmoid col |
| chr14 | 55030911 | 55217183 | SAMD4A | sigmoid col |
| chr10 | 78923221 | 79081767 | KCNMA1 | sigmoid col |
| chr13 | 97862914 | 98022524 | MBNL2 | sigmoid col |
| chr11 | 12131474 | 12271572 | MICAL2 | sigmoid col |
| chr10 | 63656893 | 63857367 | ARID5B | sigmoid col |
| chr4 | 169717184 | 169852624 | PALLD | sigmoid col |
| chr3 | 159477796 | 159629267 | IQCJ-SCHIP1,SCHIP1 | sigmoid col |
| chr10 | 125502169 | 125673984 | CPXM2 | sigmoid col |
| chr9 | 124318012 | 124544007 | DAB2IP | sigmoid col |
| chr9 | 132642101 | 132808219 | USP20,FNBP1 | sigmoid col |
| chr2 | 47181687 | 47336185 | TTC7A,C2orf61 | sigmoid col |
| chr20 | 45924428 | 46014038 | ZMYND8,LOC100131496 | sigmoid col |
| chr14 | 59646281 | 59846774 | DAAM1 | sigmoid col |
| chr3 | 64090594 | 64282175 | PRICKLE2 | sigmoid col |

| | | | | |
|---|---|---|---|---|
| chr11 | 65237402 | 65278433 | MALAT1 | sigmoid col |
| chr12 | 109023871 | 109129285 | SELPLG,CORO1C | sigmoid col |
| chr9 | 79412710 | 79522712 | PRUNE2 | sigmoid col |
| chr13 | 110966059 | 111037875 | COL4A2 | sigmoid col |
| chr1 | 92175417 | 92357108 | TGFBR3 | sigmoid col |
| chr3 | 123030630 | 123169932 | ADCY5 | sigmoid col |
| chr4 | 87919942 | 88045937 | AFF1 | sigmoid col |
| chr19 | 39125214 | 39227056 | ACTN4,CAPN12,EIF3K | sigmoid col |
| chr4 | 37953028 | 38136565 | TBC1D1,PTTG2 | sigmoid col |
| chr9 | 133639529 | 133763516 | ABL1 | sigmoid col |
| chr1 | 201847186 | 201919717 | SHISA4,LMOD1,IPO9 | sigmoid col |
| chr11 | 113979520 | 114074224 | ZBTB16 | sigmoid col |
| chr14 | 99976953 | 100072481 | CCDC85C,CCNK | sigmoid col |
| chr7 | 27199991 | 27240800 | HOXA10,HOXA9,MIR196B,HO | sigmoid col |
| chr1 | 203408496 | 203530711 | PRELP,OPTC | sigmoid col |
| chr1 | 201416945 | 201483187 | PHLDA3,CSRP1 | sigmoid col |
| chr5 | 131712779 | 131842675 | SLC22A5,C5orf56,IRF1 | sigmoid col |
| chr5 | 172259497 | 172359615 | ERGIC1 | sigmoid col |
| chr19 | 11236442 | 11321307 | KANK2,SPC24,LDLR,DOCK6 | sigmoid col |
| chr4 | 174387053 | 174497382 | NBLA00301,HAND2 | sigmoid col |
| chr7 | 4670899 | 4817053 | FOXK1,KIAA0415 | sigmoid col |
| chr2 | 43519439 | 43714382 | THADA | sigmoid col |
| chr10 | 592954 | 735712 | DIP2C,C10orf108 | sigmoid col |
| chr9 | 71030993 | 71176468 | C9orf71,PGM5 | sigmoid col |
| chr10 | 95135439 | 95243763 | MYOF | sigmoid col |
| chr17 | 38588850 | 38716694 | IGFBP4,TNS4,CCR7 | sigmoid col |
| chr2 | 128386624 | 128435773 | MYO7B,LIMS2,GPR17 | sigmoid col |
| chr16 | 17389505 | 17511947 | XYLT1 | sigmoid col |
| chr17 | 71273682 | 71366126 | CDC42EP4,SDK2 | sigmoid col |
| chr17 | 17583962 | 17697820 | SMCR5,RAI1 | sigmoid col |
| chr9 | 72654432 | 72777291 | MAMDC2 | sigmoid col |
| chr5 | 148782443 | 148819183 | MIR145,MIR143,LOC728264 | sigmoid col |
| chr16 | 85546904 | 85708470 | KIAA0182 | sigmoid col |
| chr2 | 238766868 | 238841331 | RAMP1 | sigmoid col |
| chr2 | 238569476 | 238654272 | LRRFIP1 | sigmoid col |
| chr12 | 125088063 | 125271535 | SCARB1 | sigmoid col |
| chr7 | 5366560 | 5470660 | TNRC18 | sigmoid col |
| chr18 | 8701384 | 8861049 | KIAA0802 | sigmoid col |
| chr1 | 156425635 | 156484058 | MEF2D | sigmoid col |
| chr1 | 225597192 | 225677621 | LBR,ENAH | sigmoid col |
| chr2 | 36579524 | 36743370 | CRIM1 | sigmoid col |
| chr6 | 56299611 | 56439244 | DST | sigmoid col |
| chr4 | 148652156 | 148788088 | ARHGAP10 | sigmoid col |
| chr3 | 141079405 | 141171329 | ZBTB38 | sigmoid col |
| chr9 | 132934813 | 133005625 | NCS1 | sigmoid col |
| chr20 | 43145668 | 43247483 | PKIG,SERINC3 | sigmoid col |
| chr20 | 60876684 | 60958362 | ADRM1,LAMA5 | sigmoid col |
| chr5 | 149107516 | 149211907 | PPARGC1B | sigmoid col |
| chr9 | 101704282 | 101834447 | COL15A1 | sigmoid col |
| chr19 | 1238058 | 1276973 | C19orf23,CIRBP,C19orf24,ATI | sigmoid col |
| chr7 | 101498850 | 101606055 | CUX1 | sigmoid col |
| chr16 | 1199202 | 1254782 | CACNA1H | sigmoid col |

| | | | | |
|---|---|---|---|---|
| chr1 | 2160092 | 2263332 | MORN1,SKI | sigmoid col |
| chr13 | 33742181 | 33864932 | STARD13 | sigmoid col |
| chr2 | 23724453 | 23846277 | KLHL29 | sigmoid col |
| chr2 | 20765692 | 20852503 | HS1BP3 | sigmoid col |
| chr17 | 43343399 | 43399417 | LOC100133991,MAP3K14 | sigmoid col |
| chr1 | 202466641 | 202569811 | SYT2,PPP1R12B | sigmoid col |
| chr14 | 93418611 | 93583402 | ITPK1,ITPK1-AS1 | sigmoid col |
| chr19 | 6035243 | 6110632 | RFX2 | sigmoid col |
| chr2 | 1594542 | 1762048 | PXDN | sigmoid col |
| chr11 | 118740190 | 118805385 | CXCR5,BCL9L | sigmoid col |
| chr11 | 10599218 | 10732178 | LOC100129827,MRVI1 | sigmoid col |
| chr10 | 7575867 | 7713729 | ITIH5 | sigmoid col |
| chr11 | 68764236 | 68857608 | TPCN2,MRGPRF | sigmoid col |
| chr10 | 79255372 | 79401549 | KCNMA1 | sigmoid col |
| chr9 | 672906 | 759755 | KANK1 | sigmoid col |
| chr16 | 86523531 | 86553845 | LOC400550,FOXF1 | sigmoid col |
| chr1 | 61508425 | 61670191 | NFIA | sigmoid col |
| chr9 | 129837952 | 129965851 | RALGPS1,ANGPTL2 | sigmoid col |
| chr1 | 162621556 | 162706941 | DDR2 | sigmoid col |
| chr14 | 32866796 | 32994109 | AKAP6 | sigmoid col |
| chr6 | 34981284 | 35064986 | ANKS1A | sigmoid col |
| chr11 | 73017480 | 73098013 | ARHGEF17,RELT | sigmoid col |
| chr9 | 130253859 | 130348843 | LRSAM1,FAM129B | sigmoid col |
| chr5 | 146762126 | 146893083 | STK32A,DPYSL3 | sigmoid col |
| chr3 | 194822790 | 194981545 | C3orf21 | sigmoid col |
| chr12 | 6276111 | 6347067 | CD9 | sigmoid col |
| chr12 | 122833581 | 122922866 | CLIP1 | sigmoid col |
| chr13 | 110905931 | 110965944 | COL4A2,COL4A1 | sigmoid col |
| chr9 | 90112168 | 90236181 | DAPK1 | sigmoid col |
| chr14 | 65077445 | 65231705 | SPTB,PLEKHG3 | sigmoid col |
| chr12 | 109163685 | 109253002 | SSH1 | sigmoid col |
| chr3 | 11539082 | 11686457 | VGLL4,ATG7 | sigmoid col |
| chr16 | 84851819 | 84987869 | CRISPLD2 | sigmoid col |
| chr9 | 137528580 | 137631128 | COL5A1 | sigmoid col |
| chr8 | 142126398 | 142185325 | DENND3 | sigmoid col |
| chrX | 135228332 | 135298250 | FHL1,MAP7D3 | sigmoid col |
| chr11 | 12783407 | 12937416 | TEAD1 | sigmoid col |
| chr20 | 47956869 | 48101382 | KCNB1 | sigmoid col |
| chr2 | 161124304 | 161286771 | RBMS1 | sigmoid col |
| chr6 | 151559643 | 151661227 | AKAP12 | sigmoid col |
| chr5 | 14142004 | 14221452 | TRIO | sigmoid col |
| chr17 | 79300430 | 79404230 | BAHCC1,TMEM105 | sigmoid col |
| chr15 | 63332306 | 63400991 | TPM1 | sigmoid col |
| chr22 | 41762080 | 41857565 | TEF,PHF5A,TOB2 | sigmoid col |
| chr22 | 31471398 | 31504800 | SMTN,SELM | sigmoid col |
| chr14 | 77466311 | 77519802 | C14orf4 | sigmoid col |
| chr2 | 217535404 | 217627949 | IGFBP5 | sigmoid col |
| chr11 | 94464921 | 94580027 | AMOTL1 | sigmoid col |
| chr14 | 100196453 | 100290045 | EML1 | sigmoid col |
| chr1 | 164528211 | 164644042 | PBX1 | sigmoid col |
| chr1 | 156066200 | 156114581 | LMNA | sigmoid col |
| chr8 | 124498557 | 124560709 | FBXO32 | sigmoid col |

| | | | | |
|---|---|---|---|---|
| chr7 | 28037096 | 28181652 | JAZF1 | sigmoid col |
| chr13 | 99097085 | 99234797 | FARP1,STK24 | sigmoid col |
| chr19 | 47582415 | 47714915 | SAE1,ZC3H4 | sigmoid col |
| chr6 | 112500005 | 112620707 | LAMA4 | sigmoid col |
| chr7 | 5691818 | 5750139 | RNF216 | sigmoid col |
| chr6 | 108865426 | 108944881 | FOXO3 | sigmoid col |
| chr10 | 104503553 | 104579933 | C10orf26 | sigmoid col |
| chr1 | 54734312 | 54875336 | SSBP3 | sigmoid col |
| chr12 | 94060538 | 94182137 | CRADD | sigmoid col |
| chr14 | 69244470 | 69287563 | C14orf181,ZFP36L1 | sigmoid col |
| chr7 | 115849675 | 115928979 | TES | sigmoid col |
| chr1 | 36580070 | 36673586 | MAP7D1,TRAPPC3 | sigmoid col |
| chr12 | 54783803 | 54833637 | ZNF385A,ITGA5 | sigmoid col |
| chr9 | 132213147 | 132264695 | | sigmoid col |
| chr15 | 93561282 | 93634675 | CHD2,RGMA | sigmoid col |
| chr14 | 68400418 | 68528086 | RAD51L1 | sigmoid col |
| chr18 | 57290187 | 57372921 | CCBE1 | sigmoid col |
| chr2 | 40542500 | 40686954 | SLC8A1 | sigmoid col |
| chr5 | 172158592 | 172234288 | DUSP1 | sigmoid col |
| chr11 | 117040136 | 117097967 | PCSK7,TAGLN,PAFAH1B2,SIDT | sigmoid col |
| chr11 | 68078439 | 68175071 | LRP5 | sigmoid col |
| chr15 | 37368349 | 37426866 | MEIS2 | sigmoid col |
| chr3 | 188225391 | 188452589 | LPP | sigmoid col |
| chr3 | 149244524 | 149380951 | WWTR1 | sigmoid col |
| chr11 | 125876120 | 126012385 | CDON | sigmoid col |
| chr15 | 63120449 | 63213883 | TLN2 | sigmoid col |
| chr3 | 57873230 | 57961360 | SLMAP | sigmoid col |
| chr1 | 9349430 | 9475511 | SPSB1 | sigmoid col |
| chr1 | 21619274 | 21674780 | ECE1 | sigmoid col |
| chr3 | 187921447 | 187968722 | LPP | sigmoid col |
| chr22 | 46436271 | 46467014 | C22orf26,LOC100271722,LO( | sigmoid col |
| chr4 | 141846189 | 141981686 | RNF150 | sigmoid col |
| chr5 | 123970162 | 124086007 | ZNF608 | sigmoid col |
| chr12 | 56091853 | 56126818 | BLOC1S1,CD63,RDH5,ITGA7 | sigmoid col |
| chr9 | 35487782 | 35565278 | FAM166B,RUSC2 | sigmoid col |
| chr19 | 48092751 | 48171743 | GLTSCR1 | sigmoid col |
| chr22 | 43281319 | 43405310 | PACSIN2 | sigmoid col |
| chr12 | 15268027 | 15380741 | RERG | sigmoid col |
| chr1 | 12598650 | 12683379 | DHRS3 | sigmoid col |
| chr1 | 154377066 | 154419815 | IL6R | sigmoid col |
| chr17 | 42852367 | 42922393 | GJC1,ADAM11 | sigmoid col |
| chr7 | 75920137 | 76023434 | HSPB1,YWHAG,SRCRB4D | sigmoid col |
| chr3 | 42053555 | 42143555 | TRAK1 | sigmoid col |
| chr14 | 94393756 | 94468144 | C14orf48,FAM181A,ASB2 | sigmoid col |
| chr9 | 130704493 | 130745146 | FAM102A | sigmoid col |
| chr10 | 120965252 | 121078864 | GRK5 | sigmoid col |
| chr16 | 88803495 | 88852485 | FAM38A | sigmoid col |
| chr16 | 27323992 | 27386418 | IL4R | sigmoid col |
| chr5 | 67509755 | 67587304 | PIK3R1 | sigmoid col |
| chr7 | 137592839 | 137686997 | CREB3L2 | sigmoid col |
| chr10 | 126293018 | 126434844 | FAM53B,LHPP | sigmoid col |
| chr12 | 109535862 | 109624404 | UNG,ACACB | sigmoid col |

| | | | | |
|---|---|---|---|---|
| chr3 | 124479910 | 124620347 | ITGB5 | sigmoid col |
| chr12 | 48192005 | 48224322 | HDAC7 | sigmoid col |
| chr20 | 35903313 | 35995346 | MANBAL,SRC | sigmoid col |
| chr20 | 35437506 | 35495157 | C20orf117 | sigmoid col |
| chr6 | 57035092 | 57092216 | BAG2,ZNF451,RAB23 | sigmoid col |
| chr22 | 46467107 | 46491336 | MIR3619,LOC400931 | sigmoid col |
| chr16 | 81634224 | 81765552 | CMIP | sigmoid col |
| chr17 | 76849214 | 76933001 | TIMP2 | sigmoid col |
| chr2 | 67007294 | 67142981 | | sigmoid col |
| chr1 | 204367384 | 204483013 | PPP1R15B,PIK3C2B | sigmoid col |
| chr11 | 44746231 | 44808108 | TSPAN18 | sigmoid col |
| chr17 | 40497778 | 40589509 | STAT3,PTRF | sigmoid col |
| chr10 | 123855760 | 123953009 | TACC2 | sigmoid col |
| chr19 | 16174554 | 16225919 | TPM4,RAB8A | sigmoid col |
| chr11 | 8719370 | 8758695 | ST5 | sigmoid col |
| chr6 | 57107542 | 57183112 | PRIM2 | sigmoid col |
| chr7 | 116129452 | 116203091 | CAV1,CAV2 | sigmoid col |
| chr1 | 12484439 | 12580357 | VPS13D,SNORA59A | sigmoid col |
| chr1 | 234717012 | 234755007 | IRF2BP2 | sigmoid col |
| chr11 | 65183541 | 65216947 | MIR612,NEAT1 | sigmoid col |
| chr11 | 10876226 | 10967707 | LOC729013,ZBED5 | sigmoid col |
| chr15 | 99392269 | 99508396 | IGF1R | sigmoid col |
| chr14 | 69051375 | 69157548 | RAD51L1 | sigmoid col |
| chr10 | 18428205 | 18541375 | CACNB2 | sigmoid col |
| chr7 | 107557949 | 107657750 | LAMB1,DLD | sigmoid col |
| chr13 | 47125988 | 47274875 | LRCH1 | sigmoid col |
| chr1 | 234622959 | 234702362 | | sigmoid col |
| chr15 | 96859951 | 96900823 | NR2F2,MIR1469 | sigmoid col |
| chr9 | 72997531 | 73057546 | KLF9 | sigmoid col |
| chr6 | 154747818 | 154833872 | CNKSR3 | sigmoid col |
| chr16 | 73053000 | 73117173 | ZFHX3 | sigmoid col |
| chr6 | 35435315 | 35467112 | TULP1,TEAD3,RPL10A | sigmoid col |
| chr8 | 23149584 | 23230539 | R3HCC1,LOXL2 | sigmoid col |
| chr1 | 203236313 | 203297871 | BTG2,LOC730227 | sigmoid col |
| chr8 | 11652611 | 11756002 | FDFT1,CTSB | sigmoid col |
| chr16 | 50581639 | 50662105 | NKD1 | sigmoid col |
| chr2 | 43437386 | 43506425 | THADA,LOC100129726,ZFP36 | sigmoid col |
| chr10 | 47081331 | 47149744 | LOC728643,PPYR1,LOC64365 | sigmoid col |
| chr22 | 36835145 | 36878966 | TXN2 | sigmoid col |
| chr6 | 56502175 | 56642381 | DST | sigmoid col |
| chr17 | 38239383 | 38271613 | NR1D1,THRA | sigmoid col |
| chr22 | 36223683 | 36327645 | RBFOX2 | sigmoid col |
| chr1 | 236134203 | 236232684 | NID1 | sigmoid col |
| chr12 | 122488077 | 122530621 | BCL7A,MLXIP | sigmoid col |
| chr1 | 225760700 | 225851423 | ENAH | sigmoid col |
| chr4 | 57894181 | 57986667 | LOC255130,POLR2B,IGFBP7 | sigmoid col |
| chr10 | 104341586 | 104437047 | SUFU,ARL3,TRIM8 | sigmoid col |
| chr3 | 111570158 | 111691184 | PHLDB2 | sigmoid col |
| chr10 | 79108145 | 79242237 | KCNMA1 | sigmoid col |
| chr7 | 104581435 | 104687801 | MLL5,LOC100216545 | sigmoid col |
| chr17 | 57405947 | 57485161 | YPEL2 | sigmoid col |
| chr7 | 150634953 | 150683198 | KCNH2 | sigmoid col |

| | | | | |
|---|---|---|---|---|
| chr19 | 13941672 | 13964655 | LOC284454,MIR24-2,ZSWIM4 | sigmoid col |
| chr7 | 55085526 | 55208921 | EGFR | sigmoid col |
| chr15 | 98503776 | 98569744 | ARRDC4 | sigmoid col |
| chr14 | 91073532 | 91166211 | TTC7B | sigmoid col |
| chr2 | 227586129 | 227667358 | IRS1 | sigmoid col |
| chr6 | 110641889 | 110686680 | C6orf186 | sigmoid col |
| chr4 | 119655117 | 119715604 | SEC24D | sigmoid col |
| chr19 | 15353171 | 15443983 | BRD4 | sigmoid col |
| chr15 | 67356504 | 67450304 | SMAD3 | sigmoid col |
| chr15 | 40613011 | 40636781 | C15orf52 | sigmoid col |
| chr3 | 30467238 | 30570282 | | sigmoid col |
| chr2 | 28806399 | 28941035 | PLB1 | sigmoid col |
| chr2 | 47030981 | 47110289 | LOC100134259,LOC388948 | sigmoid col |
| chr5 | 139015481 | 139091585 | CXXC5 | sigmoid col |
| chr11 | 123034074 | 123132374 | ASAM | sigmoid col |
| chr1 | 95319765 | 95403896 | LOC729970,SLC44A3,CNN3 | sigmoid col |
| chr19 | 5921146 | 5994938 | RFX2,RANBP3 | sigmoid col |
| chrX | 153575502 | 153613100 | FLNA,EMD | sigmoid col |
| chr9 | 37993416 | 38071169 | SHB | sigmoid col |
| chr17 | 2286130 | 2325648 | METT10D,LOC284009,MNT | sigmoid col |
| chr15 | 65009551 | 65070516 | RBPMS2,MIR1272 | sigmoid col |
| chr4 | 88390459 | 88465132 | SPARCL1 | sigmoid col |
| chr9 | 97805573 | 97859084 | MIR24-1,MIR23B,C9orf3,MIR2 | sigmoid col |
| chr10 | 75756886 | 75884412 | AP3M1,VCL | sigmoid col |
| chr2 | 86410137 | 86553030 | MRPL35,REEP1,IMMT | sigmoid col |
| chr12 | 4377173 | 4419299 | CCND2 | sigmoid col |
| chr20 | 10571910 | 10656582 | C20orf94,JAG1 | sigmoid col |
| chr15 | 89627766 | 89711757 | ABHD2 | sigmoid col |
| chr5 | 176881044 | 176928486 | PRR7,DBN1,PDLIM7 | sigmoid col |
| chr1 | 164715593 | 164828140 | PBX1 | sigmoid col |
| chr17 | 1325939 | 1401306 | MYO1C,CRK,INPP5K | sigmoid col |
| chr3 | 112910936 | 113011256 | WDR52,BOC | sigmoid col |
| chr17 | 36574414 | 36644029 | ARHGAP23 | sigmoid col |
| chr12 | 96581411 | 96658001 | ELK3 | sigmoid col |
| chr6 | 76108625 | 76204922 | FILIP1 | sigmoid col |
| chr19 | 39886574 | 39907815 | ZFP36,PLEKHG2,MED29 | sigmoid col |
| chr20 | 61320393 | 61393710 | NTSR1 | sigmoid col |
| chr17 | 48250244 | 48293550 | COL1A1,SGCA | sigmoid col |
| chr10 | 73722050 | 73777707 | CHST3 | sigmoid col |
| chr19 | 12868209 | 12907877 | HOOK2,PRDX2,JUNB,BEST2 | sigmoid col |
| chr10 | 112499406 | 112599709 | RBM20 | sigmoid col |
| chr5 | 172063786 | 172127676 | NEURL1B | sigmoid col |
| chr6 | 16697604 | 16772372 | ATXN1 | sigmoid col |
| chr18 | 55842632 | 55956791 | NEDD4L | sigmoid col |
| chr22 | 38274049 | 38334283 | MICALL1,EIF3L | sigmoid col |
| chr18 | 46446572 | 46502983 | SMAD7 | sigmoid col |
| chr9 | 139404505 | 139471332 | NOTCH1 | sigmoid col |
| chr10 | 35616549 | 35750728 | CCNY | sigmoid col |
| chr14 | 53328578 | 53428011 | FERMT2 | sigmoid col |
| chr14 | 70076712 | 70194335 | KIAA0247 | sigmoid col |
| chr8 | 124629210 | 124720848 | KLHL38,ANXA13 | sigmoid col |
| chr17 | 78988848 | 79072229 | BAIAP2,FLJ90757 | sigmoid col |

| | | | | |
|---|---|---|---|---|
| chr17 | 57881455 | 57934720 | MIR21,TMEM49 | sigmoid col |
| chr17 | 76309768 | 76416976 | SOCS3,PGS1 | sigmoid col |
| chr11 | 12692301 | 12768861 | TEAD1 | sigmoid col |
| chr6 | 111860433 | 111931087 | TRAF3IP2,LOC643749 | sigmoid col |
| chr13 | 21564071 | 21654663 | LATS2 | sigmoid col |
| chr17 | 38440173 | 38514852 | RARA,CDC6 | sigmoid col |
| chr8 | 26433372 | 26517506 | DPYSL2 | sigmoid col |
| chr11 | 2387145 | 2441934 | TSSC4,CD81,TRPM5 | sigmoid col |
| chr19 | 3632861 | 3700511 | PIP5K1C | sigmoid col |
| chr4 | 37861848 | 37933881 | PGM2,TBC1D1 | sigmoid col |
| chr2 | 109149701 | 109254763 | LIMS1 | sigmoid col |
| chr5 | 58978003 | 59096098 | PDE4D | sigmoid col |
| chr10 | 521725 | 576032 | DIP2C | sigmoid col |
| chr2 | 207979968 | 208033795 | KLF7 | sigmoid col |
| chr22 | 18371559 | 18488948 | MIR648,MICAL3 | sigmoid col |
| chr2 | 218220948 | 218305822 | DIRC3 | sigmoid col |
| chr3 | 187968830 | 187999483 | LPP | sigmoid col |
| chr2 | 20577957 | 20667975 | RHOB | sigmoid col |
| chr7 | 105409942 | 105548294 | ATXN7L1 | sigmoid col |
| chr1 | 31348445 | 31402303 | SDC3 | sigmoid col |
| chr8 | 103588289 | 103675843 | KLF10 | sigmoid col |
| chr10 | 134371726 | 134458649 | INPP5A | sigmoid col |
| chr4 | 151486788 | 151520708 | MAB21L2,LRBA | sigmoid col |
| chr12 | 119612670 | 119658413 | HSPB8 | sigmoid col |
| chr1 | 85986921 | 86078803 | CYR61,DDAH1 | sigmoid col |
| chr9 | 138947298 | 139002807 | NACC2 | sigmoid col |
| chr6 | 7106520 | 7189987 | RREB1 | sigmoid col |
| chr16 | 75075893 | 75146308 | LDHD,ZNRF1 | sigmoid col |
| chr5 | 171826882 | 171883748 | SH3PXD2B | sigmoid col |
| chr10 | 101278938 | 101311740 | NKX2-3 | sigmoid col |
| chr9 | 14237944 | 14327352 | NFIB | sigmoid col |
| chr20 | 56007710 | 56070969 | | sigmoid col |
| chr12 | 16721910 | 16767187 | LMO3 | sigmoid col |
| chr2 | 218111207 | 218205163 | DIRC3 | sigmoid col |
| chr11 | 64617001 | 64655859 | EHD1 | sigmoid col |
| chr1 | 150118651 | 150138830 | PLEKHO1 | sigmoid col |
| chr10 | 104153197 | 104171320 | NFKB2,PSD | sigmoid col |
| chr10 | 29903485 | 29942368 | SVIL | sigmoid col |
| chr20 | 48702182 | 48790076 | TMEM189,UBE2V1,TMEM189- | sigmoid col |
| chr2 | 60726799 | 60787299 | BCL11A | sigmoid col |
| chr7 | 50719236 | 50798934 | GRB10 | sigmoid col |
| chr3 | 30638222 | 30735636 | TGFBR2 | sigmoid col |
| chr11 | 65042683 | 65098074 | CDC42EP2,POLA2 | sigmoid col |
| chr3 | 99551057 | 99602185 | FILIP1L,C3orf26,MIR548G | sigmoid col |
| chr8 | 38261736 | 38326827 | LETM2,FGFR1 | sigmoid col |
| chr17 | 74676773 | 74706864 | MXRA7 | sigmoid col |
| chr1 | 19220787 | 19289575 | ALDH4A1,IFFO2 | sigmoid col |
| chr1 | 157961886 | 158019800 | KIRREL | sigmoid col |
| chr3 | 50264320 | 50311383 | GNAI2,SEMA3B | sigmoid col |
| chr1 | 8063343 | 8158430 | ERRFI1 | sigmoid col |
| chr1 | 2120414 | 2147468 | LOC100128003,C1orf86 | sigmoid col |
| chr12 | 27390901 | 27478271 | STK38L | sigmoid col |

| | | | | |
|---|---|---|---|---|
| chr14 | 68972191 | 69031834 | RAD51L1 | sigmoid col |
| chr10 | 79596279 | 79680812 | DLG5 | sigmoid col |
| chr7 | 158570050 | 158623810 | ESYT2 | sigmoid col |
| chr1 | 115580505 | 115643569 | TSPAN2 | sigmoid col |
| chr7 | 120627294 | 120690303 | C7orf58 | sigmoid col |
| chr12 | 109881244 | 109916175 | UBE3B,MYO1H,KCTD10 | sigmoid col |
| chr20 | 25202859 | 25292786 | PYGB,ENTPD6,ABHD12 | sigmoid col |
| chr14 | 90846545 | 90886471 | CALM1 | sigmoid col |
| chr17 | 45301627 | 45395406 | ITGB3 | sigmoid col |
| chr1 | 145433959 | 145458236 | TXNIP,POLR3GL | sigmoid col |
| chr9 | 133022438 | 133071058 | | sigmoid col |
| chr8 | 120959030 | 121054329 | DEPDC6 | sigmoid col |
| chr1 | 85614932 | 85711936 | SYDE2 | sigmoid col |
| chr10 | 93329862 | 93395272 | PPP1R3C,LOC100188947 | sigmoid col |
| chr3 | 48029903 | 48131561 | MAP4 | sigmoid col |
| chr2 | 28611250 | 28643133 | FOSL2 | sigmoid col |
| chr10 | 75595628 | 75649631 | CAMK2G | sigmoid col |
| chr14 | 102236604 | 102321184 | PPP2R5C | sigmoid col |
| chr11 | 68685990 | 68736700 | IGHMBP2 | sigmoid col |
| chr6 | 10545317 | 10628249 | GCNT2 | sigmoid col |
| chr14 | 103981067 | 104013232 | CKB,TRMT61A | sigmoid col |
| chr7 | 2756323 | 2837050 | GNA12 | sigmoid col |
| chr1 | 150530941 | 150553244 | ADAMTSL4,MCL1 | sigmoid col |
| chr4 | 187586792 | 187648675 | FAT1 | sigmoid col |
| chr1 | 221870972 | 221917321 | DUSP10 | sigmoid col |
| chr19 | 48184596 | 48249834 | GLTSCR1,GLTSCR2,EHD2 | sigmoid col |
| chr7 | 30877038 | 30972025 | AQP1,INMT-FAM188B,FAM18 | sigmoid col |
| chr11 | 8775643 | 8813607 | ST5 | sigmoid col |
| chr20 | 31034326 | 31073948 | C20orf112 | sigmoid col |
| chr10 | 121238169 | 121335196 | RGS10,TIAL1 | sigmoid col |
| chr5 | 148757547 | 148773534 | IL17B | sigmoid col |
| chr14 | 69504815 | 69574740 | DCAF5 | sigmoid col |
| chr15 | 90576757 | 90656152 | ZNF710,IDH2 | sigmoid col |
| chr18 | 9093618 | 9148138 | NDUFV2,ANKRD12 | sigmoid col |
| chr2 | 216516186 | 216589576 | LOC646324 | sigmoid col |
| chr9 | 134247926 | 134312421 | BAT2L1 | sigmoid col |
| chr17 | 48968703 | 49045988 | SPAG9 | sigmoid col |
| chr1 | 182990044 | 183071462 | LAMC1 | sigmoid col |
| chr11 | 74380735 | 74443171 | CHRDL2 | sigmoid col |
| chr3 | 45980573 | 46049078 | CXCR6,FYCO1 | sigmoid col |
| chr7 | 93976981 | 94055470 | COL1A2 | sigmoid col |
| chr17 | 63002040 | 63076267 | GNA13 | sigmoid col |
| chr10 | 34990359 | 35105809 | PARD3 | sigmoid col |
| chr11 | 6613324 | 6637853 | TPP1,ILK,RRP8,TAF10 | sigmoid col |
| chr16 | 22199619 | 22253252 | EEF2K | sigmoid col |
| chr1 | 68633839 | 68699207 | WLS,MIR1262 | sigmoid col |
| chr7 | 73865751 | 73929299 | GTF2IRD1 | sigmoid col |
| chr20 | 37554346 | 37617249 | FAM83D,DHX35 | sigmoid col |
| chr12 | 26844187 | 26984555 | ITPR2 | sigmoid col |
| chr18 | 72908650 | 72940424 | ZADH2,TSHZ1 | sigmoid col |
| chr18 | 74147317 | 74210194 | ZNF516 | sigmoid col |
| chr2 | 48735300 | 48800658 | STON1,STON1-GTF2A1L,KLRA( | sigmoid col |

| | | | | |
|---|---|---|---|---|
| chr3 | 71244658 | 71321149 | FOXP1 | sigmoid col |
| chr10 | 134260591 | 134358926 | INPP5A,C10orf91 | sigmoid col |
| chr4 | 80917914 | 80996173 | ANTXR2 | sigmoid col |
| chr10 | 121409580 | 121446709 | BAG3 | sigmoid col |
| chr9 | 130805425 | 130872010 | SLC25A25,NAIF1 | sigmoid col |
| chr10 | 61594492 | 61669700 | CCDC6 | sigmoid col |
| chr2 | 232461634 | 232509602 | | sigmoid col |
| chr2 | 9305413 | 9404895 | ASAP2 | sigmoid col |
| chr22 | 44699369 | 44760760 | KIAA1644 | sigmoid col |
| chr10 | 21798563 | 21825442 | MLLT10,C10orf140 | sigmoid col |
| chr3 | 177156282 | 177209533 | | sigmoid col |
| chr19 | 46266780 | 46289665 | SIX5,DMPK,DMWD | sigmoid col |
| chr11 | 119168414 | 119212304 | CBL,MCAM,RNF26,C1QTNF5 | sigmoid col |
| chr2 | 37867865 | 37918020 | CDC42EP3 | sigmoid col |
| chr17 | 38213871 | 38237889 | THRA | sigmoid col |
| chr19 | 17186542 | 17256567 | MYO9B | sigmoid col |
| chr8 | 59956982 | 60033412 | TOX | sigmoid col |
| chr3 | 69786901 | 69842232 | MITF | sigmoid col |
| chr15 | 65127482 | 65197933 | PLEKHO2 | sigmoid col |
| chr9 | 125108325 | 125169166 | PTGS1 | sigmoid col |
| chr1 | 8175372 | 8235316 | | sigmoid col |
| chr11 | 75012502 | 75064289 | ARRB1,MIR326 | sigmoid col |
| chr3 | 151974071 | 152003996 | LOC401093,MBNL1 | sigmoid col |
| chr12 | 6431171 | 6453746 | PLEKHG6,TNFRSF1A | sigmoid col |
| chr11 | 75258793 | 75296382 | SERPINH1 | sigmoid col |
| chr9 | 12774502 | 12827397 | C9orf150 | sigmoid col |
| chr17 | 17711956 | 17754247 | MIR33B,SREBF1,TOM1L2,RAI1 | sigmoid col |
| chr12 | 53431738 | 53469153 | SPRYD3,LOC283335,TENC1,EI | sigmoid col |
| chr7 | 128457839 | 128503963 | CCDC136,FLNC,ATP6V1F | sigmoid col |
| chr20 | 55963562 | 55991450 | RBM38 | sigmoid col |
| chr1 | 25030875 | 25133651 | CLIC4 | sigmoid col |
| chr4 | 15002301 | 15045845 | CPEB2 | sigmoid col |
| chr17 | 55149355 | 55192078 | AKAP1 | sigmoid col |
| chr7 | 27134872 | 27161084 | HOXA1,HOXA2,HOXA3 | sigmoid col |
| chr1 | 15472102 | 15542662 | TMEM51,C1orf126 | sigmoid col |
| chr12 | 47458892 | 47499194 | AMIGO2 | sigmoid col |
| chr15 | 39718874 | 39797713 | | sigmoid col |
| chr17 | 55935568 | 55960848 | CUEDC1 | sigmoid col |
| chr11 | 122927309 | 123016970 | HSPA8,ASAM | sigmoid col |
| chrX | 2582021 | 2642515 | CD99 | sigmoid col |
| chr4 | 175025154 | 175079913 | | sigmoid col |
| chr18 | 9797450 | 9885275 | RAB31 | sigmoid col |
| chr12 | 26110101 | 26186440 | RASSF8 | sigmoid col |
| chr10 | 3784049 | 3830388 | KLF6 | sigmoid col |
| chr4 | 38663000 | 38699977 | FLJ13197,KLF3 | sigmoid col |
| chr12 | 125388500 | 125426128 | UBC | sigmoid col |
| chr11 | 117720480 | 117760101 | FXYD6 | sigmoid col |
| chr9 | 36135788 | 36167072 | GLIPR2 | sigmoid col |
| chr12 | 68688635 | 68759780 | MDM1 | sigmoid col |
| chr7 | 27161291 | 27193446 | HOXA7,HOXA5,HOXA6,HOXA4 | sigmoid col |
| chr22 | 34181247 | 34283438 | LARGE | sigmoid col |
| chr6 | 53152084 | 53226111 | ELOVL5 | sigmoid col |

| | | | | |
|---|---|---|---|---|
| chr12 | 2160447 | 2187916 | CACNA1C | sigmoid col |
| chr2 | 46038237 | 46118074 | PRKCE | sigmoid col |
| chr12 | 7032801 | 7056201 | ENO2,PTPN6,ATN1,C12orf57 | sigmoid col |
| chr15 | 70339787 | 70415515 | MIR629,TLE3 | sigmoid col |
| chr12 | 120661372 | 120703574 | PXN | sigmoid col |
| chr3 | 145834922 | 145896384 | PLOD2 | sigmoid col |
| chr1 | 206845718 | 206924160 | MAPKAPK2 | sigmoid col |
| chr1 | 221046494 | 221070203 | HLX | sigmoid col |
| chr1 | 32029924 | 32059131 | TINAGL1 | sigmoid col |
| chr19 | 4038773 | 4078893 | ZBTB7A | sigmoid col |
| chr5 | 66528676 | 66612324 | | sigmoid col |
| chr19 | 13259829 | 13293234 | STX10,IER2 | sigmoid col |
| chr4 | 156579826 | 156628010 | GUCY1A3 | sigmoid col |
| chr20 | 44033191 | 44095558 | PIGT,DBNDD2,SYS1-DBNDD2 | sigmoid col |
| chr16 | 85179947 | 85272599 | LOC400548 | sigmoid col |
| chr2 | 42272369 | 42295722 | PKDCC | sigmoid col |
| chr13 | 111038226 | 111061147 | COL4A2 | sigmoid col |
| chr11 | 65331945 | 65362100 | FAM89B,EHBP1L1,KCNK7,SSS | sigmoid col |
| chr14 | 90976225 | 91047813 | TTC7B | sigmoid col |
| chr1 | 226964985 | 227016279 | | sigmoid col |
| chr20 | 21130366 | 21183811 | PLK1S1 | sigmoid col |
| chr3 | 48440804 | 48478254 | PLXNB1,CCDC51 | sigmoid col |
| chrX | 39920805 | 39969960 | BCOR | sigmoid col |
| chr9 | 116216141 | 116322303 | RGS3 | sigmoid col |
| chr20 | 35143959 | 35182009 | DLGAP4,MYL9 | sigmoid col |
| chr3 | 134068340 | 134094769 | AMOTL2 | sigmoid col |
| chr22 | 30667159 | 30745349 | TBC1D10A,SF3A1,GATSL3 | sigmoid col |
| chr5 | 171950336 | 171997826 | | sigmoid col |
| chr7 | 80451505 | 80537804 | SEMA3C | sigmoid col |
| chr3 | 156760622 | 156830552 | LOC100498859,LEKR1,LOC33 | sigmoid col |
| chr1 | 226733295 | 226796544 | C1orf95 | sigmoid col |
| chr1 | 17853251 | 17918151 | ARHGEF10L | sigmoid col |
| chr22 | 20861218 | 20924763 | MED15 | sigmoid col |
| chr1 | 32131404 | 32182175 | COL16A1 | sigmoid col |
| chr18 | 46064549 | 46124613 | KIAA0427 | sigmoid col |
| chr14 | 75724058 | 75782444 | FOS | sigmoid col |
| chr16 | 20861350 | 20925989 | LYRM1,DCUN1D3 | sigmoid col |
| chr22 | 23505338 | 23632799 | FBXW4P1,RAB36,BCR | sigmoid col |
| chr16 | 24696754 | 24806847 | TNRC6A | sigmoid col |
| chr3 | 73434490 | 73507608 | PDZRN3 | sigmoid col |
| chr16 | 56267154 | 56312569 | GNAO1,MIR3935 | sigmoid col |
| chr4 | 126234874 | 126324122 | FAT4 | sigmoid col |
| chr10 | 31015855 | 31075970 | | sigmoid col |
| chr12 | 65058493 | 65091342 | RASSF3 | sigmoid col |
| chr12 | 2068546 | 2092578 | DCP1B | sigmoid col |
| chr12 | 6640594 | 6667777 | GAPDH,NOP2,NCAPD2,IFFO1 | sigmoid col |
| chr1 | 27319790 | 27360493 | TRNP1,FAM46B | sigmoid col |
| chr7 | 836592 | 885101 | SUN1 | sigmoid col |
| chr11 | 71697296 | 71757996 | IL18BP,NUMA1,RNF121 | sigmoid col |
| chr16 | 85382882 | 85431149 | | sigmoid col |
| chr9 | 132864347 | 132907456 | GPR107 | sigmoid col |
| chr17 | 53342254 | 53380890 | HLF | sigmoid col |

| | | | | |
|---|---|---|---|---|
| chr6 | 44170549 | 44206742 | SLC29A1 | sigmoid col |
| chr11 | 74316542 | 74363135 | POLD3 | sigmoid col |
| chr16 | 87395900 | 87428140 | FBXO31,MAP1LC3B | sigmoid col |
| chr13 | 44865672 | 44907844 | | sigmoid col |
| chr10 | 101665791 | 101770972 | NCRNA00093,DNMBP | sigmoid col |
| chr12 | 80264451 | 80341648 | PPP1R12A | sigmoid col |
| chr10 | 30113983 | 30136339 | | sigmoid col |
| chr15 | 49713814 | 49764730 | FGF7,C15orf33 | sigmoid col |
| chr13 | 51796464 | 51867027 | FAM124A | sigmoid col |
| chr13 | 110872253 | 110904774 | COL4A1 | sigmoid col |
| chr7 | 5592387 | 5678098 | RNF216,FSCN1 | sigmoid col |
| chr3 | 151909717 | 151943487 | | sigmoid col |
| chr17 | 75299951 | 75330415 | 09-Sep | sigmoid col |
| chr8 | 42316065 | 42371823 | SLC20A2 | sigmoid col |
| chr1 | 208314564 | 208414558 | PLXNA2 | sigmoid col |
| chr5 | 37805863 | 37842233 | GDNF | sigmoid col |
| chr2 | 23624540 | 23705454 | KLHL29 | sigmoid col |
| chr15 | 74273687 | 74346268 | PML,STOML1 | sigmoid col |
| chr12 | 96732602 | 96796107 | CDK17 | sigmoid col |
| chr12 | 46745599 | 46798912 | SLC38A2 | sigmoid col |
| chr17 | 29864987 | 29924399 | RAB11FIP4,MIR193A,MIR365- | sigmoid col |
| chr3 | 120131889 | 120172057 | FSTL1 | sigmoid col |
| chr7 | 143064818 | 143091346 | ZYX,EPHA1 | sigmoid col |
| chr3 | 64627257 | 64676008 | ADAMTS9 | sigmoid col |
| chr6 | 35685620 | 35702787 | FKBP5,LOC285847 | sigmoid col |
| chr12 | 94195320 | 94223507 | CRADD | sigmoid col |
| chr5 | 131589843 | 131611217 | PDLIM4 | sigmoid col |
| chr9 | 75511580 | 75571215 | ALDH1A1 | sigmoid col |
| chr8 | 93063087 | 93121119 | RUNX1T1 | sigmoid col |
| chr22 | 28238691 | 28289216 | PITPNB | sigmoid col |
| chr9 | 35678406 | 35702909 | TPM2,CA9,TLN1 | sigmoid col |
| chr21 | 46494555 | 46589505 | ADARB1 | sigmoid col |
| chr8 | 28189593 | 28270744 | PNOC,ZNF395 | sigmoid col |

| |
|---|
| |
| |
| |
| |
| |
| |
| |
| |
| |
| |
| |
| |
| |
| |
| |
| |
| |
| |
| |
| |
| |
| |
| |
| |
| |
| |
| |
| |
| |
| |
| |
| |
| |
| |
| on |
| on |
| on |
| on |
| on |
| on |
| on |
| on |
| on |
| on |
| on |
| on |
| on |
| on |
| on |
| on |
| on |

| |
|---|
| on |
| on |
| on |
| on |
| on |
| on |
| on |
| on |
| on |
| on |
| on |
| on |
| on |
| on |
| on |
| on |
| on |
| on |
| on |
| on |
| on |
| on |
| on |
| on |
| on |
| on |
| on |
| on |
| on |
| on |
| on |
| on |
| on |
| on |
| on |
| on |
| on |
| on |
| on |
| on |
| on |
| on |
| on |
| on |
| on |
| on |
| on |
| on |
| on |
| on |
| on |
| on |

| |
|---|
| on |
| on |
| on |
| on |
| on |
| on |
| on |
| on |
| on |
| on |
| on |
| on |
| on |
| on |
| on |
| on |
| on |
| on |
| on |
| on |
| on |
| on |
| on |
| on |
| on |
| on |
| on |
| on |
| on |
| on |
| on |
| on |
| on |
| on |
| on |
| on |
| on |
| on |
| on |
| on |
| on |
| on |
| on |
| on |
| on |
| on |
| on |
| on |
| on |
| on |
| on |
| on |

| |
|---|
| on |
| on |
| on |
| on |
| on |
| on |
| on |
| on |
| on |
| on |
| on |
| on |
| on |
| on |
| on |
| on |
| on |
| on |
| on |
| on |
| on |
| on |
| on |
| on |
| on |
| on |
| on |
| on |
| on |
| on |
| on |
| on |
| on |
| on |
| on |
| on |
| on |
| on |
| on |
| on |
| on |
| on |
| on |
| on |
| on |
| on |
| on |
| on |
| on |
| on |
| on |
| on |

| |
|---|
| on |
| on |
| on |
| on |
| on |
| on |
| on |
| on |
| on |
| on |
| on |
| on |
| on |
| on |
| on |
| on |
| on |
| on |
| on |
| on |
| on |
| on |
| on |
| on |
| on |
| on |
| on |
| on |
| on |
| on |
| on |
| on |
| on |
| on |
| on |
| on |
| on |
| on |
| on |
| on |
| on |
| on |
| on |
| on |
| on |
| on |
| on |
| on |
| on |
| on |
| on |
| on |

| |
|---|
| on |
| on |
| on |
| on |
| on |
| on |
| on |
| on |
| on |
| on |
| on |
| on |
| on |
| on |
| on |
| on |
| on |
| on |
| on |
| on |
| on |
| on |
| on |
| on |
| on |
| on |
| on |
| on |
| on |
| on |
| on |
| on |
| on |
| on |
| on |
| on |
| on |
| on |
| on |
| on |
| on |
| on |
| on |
| on |
| on |
| on |
| on |
| on |
| on |
| on |
| on |
| on |

| |
|---|
| on |
| on |
| on |
| on |
| on |
| on |
| on |
| on |
| on |
| on |
| on |
| on |
| on |
| on |
| on |
| on |
| on |
| on |
| on |
| on |
| on |
| on |
| on |
| on |
| on |
| on |
| on |
| on |
| on |
| on |
| on |
| on |
| on |
| on |
| on |
| on |
| on |
| on |
| on |
| on |
| on |
| on |
| on |
| on |
| on |
| on |
| on |
| on |
| on |
| on |
| on |
| on |

| |
|---|
| on |
| on |
| on |
| on |
| on |
| on |
| on |
| on |
| on |
| on |
| on |
| on |
| on |
| on |
| on |
| on |
| on |
| on |
| on |
| on |
| on |
| on |
| on |
| on |
| on |
| on |
| on |
| on |
| on |
| on |
| on |
| on |
| on |
| on |
| on |
| on |
| on |
| on |
| on |
| on |
| on |
| on |
| on |
| on |
| on |
| on |
| on |
| on |
| on |
| on |
| on |
| on |

| |
|---|
| on |
| on |
| on |
| on |
| on |
| on |
| on |
| on |
| on |
| on |
| on |
| on |
| on |
| on |
| on |
| on |
| on |
| on |
| on |
| on |
| on |
| on |
| on |
| on |
| on |
| on |
| on |
| on |
| on |
| on |
| on |
| on |
| on |
| on |
| on |
| on |
| on |
| on |
| on |
| on |
| on |
| on |
| on |
| on |
| on |
| on |
| on |
| on |
| on |
| on |
| on |
| on |

| |
|---|
| on |
| on |
| on |
| on |
| on |
| on |
| on |
| on |
| on |
| on |
| on |
| on |
| on |
| on |
| on |
| on |
| on |
| on |
| on |
| on |
| on |
| on |
| on |
| on |
| on |
| on |
| on |
| on |
| on |
| on |
| on |
| on |
| on |
| on |
| on |
| on |
| on |
| on |
| on |
| on |
| on |
| on |
| on |
| on |
| on |
| on |
| on |
| on |
| on |
| on |
| on |
| on |

| |
|---|
| on |
| on |
| on |
| on |
| on |
| on |
| on |
| on |
| on |
| on |
| on |
| on |
| on |
| on |
| on |
| on |
| on |
| on |
| on |
| on |
| on |
| on |
| on |
| on |
| on |
| on |
| on |
| on |
| on |
| on |
| on |
| on |
| on |
| on |
| on |
| on |
| on |
| on |
| on |
| on |
| on |
| on |
| on |
| on |
| on |
| on |
| on |
| on |
| on |
| on |
| on |
| on |

| |
|---|
| on |
| on |
| on |
| on |
| on |
| on |
| on |
| on |
| on |
| on |
| on |
| on |
| on |
| on |
| on |
| on |
| on |
| on |
| on |
| on |
| on |
| on |
| on |
| on |
| on |
| on |
| on |
| on |
| on |
| on |
| on |
| on |
| on |

-2324
