# Supplementary material for: Recurrent Superenhancer of the Oncogene POU5F1B in Colorectal Cancers
Source: Biomed Res Int. 2021 Dec 11;2021:5405060. doi: 10.1155/2021/5405060 (PMC8684575; doi:10.1155/2021/5405060)
Supplement: Supplementary 2 — Supplementary Table 2: PCR primers. [file 5405060.f2.pdf]

	Forward Primer	Reverse Primer
Chip-qPCR	5'-GTGTATCTTAGTTCAACCAAATTGTAATCATCTG-3'	5'-CAGTGCAGGGTCCGAGGT-3'
POU5F1B	5'-CCTGAAGCAGAAGAGGATCACC-3'	5'-AAGCGGCAGATGGTCTTTTGGC-3'
GAPDH	5'-GAAGGTGAAGGTCGGAGTC-3'	5'-GAAGATGGTGATGGGATTTC-3'
